# Quality of life of Bahraini women with breast cancer: a cross sectional study

**DOI:** 10.1186/1471-2407-13-212

**Published:** 2013-04-28

**Authors:** Ghufran Ahmed Jassim, David L Whitford

**Affiliations:** 1 Royal College of Surgeons in Ireland-Medical University of Bahrain, PO Box 15503, Adliya, Bahrain

**Keywords:** Breast cancer, Bahrain, Quality of life, Bahraini women, Middle East, EORTC

## Abstract

**Background:**

Breast cancer can impact survivors in many aspects of their life. Scarce information is currently available on the quality of life of cancer survivors in Bahrain. The objective of this study is to describe the quality of life of Bahraini women with breast cancer and its association with their sociodemographic and clinical data.

**Methods:**

This is a cross sectional study in which the European Organization for Research and Treatment of Cancer Quality of Life Cancer Specific version translated into Arabic was administered to a random sample of 337 Bahraini women with breast cancer. Relevant descriptive statistics were computed for all items. The equality of means across the categories of each categorical independent variable was tested using parametric tests (ANOVA and independent t-test) or non-parametric tests (Kruskal Wallis and Mann Whitney tests) of association where appropriate.

**Results:**

Of the total sample, 239 consented to participation. The mean and median age of participants were 50.2 (SD ± 11.1) and 48.0 respectively. Participants had a mean score for global health of 63.9 (95% CI 61.21-66.66). Among functional scales, social functioning scored the highest (Mean 77.5 [95% CI 73.65-81.38]) whereas emotional functioning scored the lowest (63.4 [95% CI 59.12-67.71]). The most distressing symptom on the symptom scales was fatigability (Mean 35.2 [95% CI 31.38-39.18]). Using the disease specific tool it was found that sexual functioning scored the lowest (Mean 25.9 [95% CI 70.23-77.90]). On the symptom scale, upset due to hair loss scored the highest (Mean 46.3 [95% CI 37.82-54.84]). Significant mean differences were noted for many functional and symptom scales.

**Conclusion:**

Bahraini breast cancer survivors reported favorable overall global quality of life. Factors associated with a major reduction in all domains of quality of life included the presence of metastases, having had a mastectomy as opposed to a lumpectomy and a shorter time elapsed since diagnosis. Poorest functioning was noted in the emotional and sexual domains. The most bothersome symptoms were fatigability, upset due to hair loss and arm symptoms. This study identifies the categories of women at risk of poorer quality of life after breast cancer and the issues that most need to be addressed in this Middle East society.

## Background

Breast cancer is the most common cancer among women worldwide. It accounts for 23% of all new cancers in women excluding cancers of the skin [1,2]. Breast cancer is ranked as the most prevalent cancer among women in the Arab world [1]. Advances in diagnostic and treatment modalities have also resulted in increased survival. Thus, coping with breast cancer as a chronic disease is becoming a more common phenomenon.

In the Arab world, surgeons and oncologists dealing with breast cancer tend to believe that it presents at an earlier age with a more advanced stage at presentation [3]. This impression is particularly evident in Bahrain and other Gulf Cooperation Council [4] countries where women aged less than 40 years make up a larger percentage of total breast cancer cases than do their counterparts in Western countries [5,6]. In addition, Bahraini women similar to other Arab women face cultural taboos surrounding breast cancer. Some families fear that their daughters will not be able to marry if a mother’s diagnosis of breast cancer becomes known [7].

The increased survival of breast cancer patients, the younger age at diagnosis, and the unique set of cultural norms and values all suggest that information on quality of life (QoL) in this region may be specific and therefore important to both health care providers and patients. However, little information of this nature is available. This study aims to describe the quality of life of Bahraini women with breast cancer and to identify factors that may facilitate improvements in health care for breast cancer survivors in Bahrain.

## Methods

### Setting

Bahrain consists of an archipelago of islands located in the Arabian Gulf. It has a population of 1,106,509, of which 537,719(48.6%) are Bahrainis [8]. Bahrain has a national health service with care being free at the point of contact for Bahraini citizens. The main governmental hospital is Salmaniya Medical Complex which hosts the only oncology center in the country [4]. All diagnosed breast cancer cases are referred to this center for further adjuvant treatments.

### Sampling and recruitment

This is a descriptive cross sectional study. Bahraini women survivors of breast cancer diagnosed between 1^st^ January 1999 and 31^st^ December 2008 were identified from the Bahrain Cancer Registry. Non Bahraini women were excluded on the basis that quality of life may differ across different cultures and ethnic groupings. A sample size of 337 subjects was calculated to give 85% power at 5% significance with an estimated non response rate of 20%. A simple random sample was drawn from the Bahrain Cancer Registry using computer software. The researcher conducted a 10-minute interview with the participants after obtaining their consent in writing.

### Study instruments

A structured questionnaire collecting sociodemographic data, clinical information and quality of life data was used. Time elapsed since diagnosis was defined as: early after diagnosis (≤1 year since diagnosis), transitional period (>1 and ≤5years since diagnosis) and long term survivors (>5 to ≤10 years). Quality of life was assessed using the European Organization for Research and Treatment of Cancer QoL Cancer Specific Version (EORTC QLQ-C30, v.3.0) translated into Arabic and validated [9,10]. Items explored by the EORTC QLQ-30 include nine domains: global health, physical, role, emotional, cognitive, social functioning, fatigue, nausea/vomiting, pain, and financial impact. We also used the QoL Breast Cancer Specific Version [9,10] translated into Arabic. The EORTC QLQ-BR23 incorporates five domains: therapy side effects, arm symptoms, breast symptoms, body image, and sexual functioning.

Scores for these questions range between 0 and 100. For scales evaluating global health and function, a higher score represents higher level of functioning and health. For scales evaluating symptoms, a higher score indicates more problems and higher level of symptoms.

### Ethical consideration

Ethical approval was obtained from the ethics committees in the Royal College of Surgeons in Ireland-Medical University of Bahrain and the Ministry of Health in Bahrain.

### Statistical analysis

A supplemental scoring manual is provided with the questionnaire which was followed in the analysis [11]. The collected data were coded, entered and analyzed using the statistical package SPSS version 15.0. Relevant descriptive statistics were computed for all items. A higher score represents a “better” level of functioning, or a “worse” level of symptoms. The “Score” served as the dependent variable in the study for the purpose of data analyses. Sociodemographic data, cancer and treatment information represented the independent variables.

The equality of means across the categories of each categorical independent variable was tested using parametric tests (ANOVA and independent t-test). Non-parametric tests (Kruskal Wallis and Mann Whitney tests) were used if the statistical assumptions of using the parametric tests were violated. Additional exploration of the differences among means was determined by post hoc analysis.

As recommended by an empirical population based study [12], for the functional scales and the global quality of life, we defined subjects with problematic functioning as those who scored <33.3%, while subjects in good condition scored ≥66.7%. For symptom scales, subjects scoring < 33.3% were judged as having less severe symptoms, while those scoring ≥ 66.7% had more intense symptoms.

Linear Regression Modeling was used to build a predictive model to assess the significance of predictors and to compute the coefficient of determination. Global health, physical, emotional, cognitive and social functioning scores served as the dependent variables. All independent variables (age, duration since diagnosis, marital status, educational level, employment status, income, menopausal status, co-morbidities, pathological staging, history of metastases, lumpectomy, mastectomy, lymph node dissection , radiotherapy, hormonal therapy and chemotherapy) were categorized into two (yes and No) categories and served as predictors for the models. R squared was computed. A P-value <0.05 was considered statistically significant.

## Results

Details of 337 Bahraini women with breast cancer were taken from the Bahrain Cancer Registry. Among the sample taken from the registry 18 had died and 66 were inaccessible. Seven were ineligible due to language barrier, deafness or wrong diagnosis, and seven refused to participate (reasons for not participating were lack of time, extreme anxiety, unwillingness to share their experience or not wanting to be reminded of their experience with cancer). Two hundred and thirty nine women consented to participate and were interviewed by the researcher (recruitment rate 71%).

### Characteristics of the study sample

The characteristics of the sample are presented in Table 1. The mean and median ages of participants were 50.2 (SD ± 11.1) and 48.0 years respectively. Mean time elapsed since diagnosis was 4.22 (SD ± 2.69) years.

**Table 1:** Characteristics of the study population N = 239

**Characteristic**	**No.**	**%**
**Age n = 239**		
**≤50 year**	**137**	**57.3**
**>50 years**	**102**	**42.7**
**Time since diagnosis n = 239**		
**Early diagnosed**	**35**	**14.6**
**Transitional period**	**128**	**53.6**
**Long term survivors**	**76**	**31.8**
**Educational level n = 236**		
**Illiterate**	**60**	**25.4**
**Primary**	**30**	**12.7**
**Intermediate**	**23**	**9.7**
**High school/diploma**	**81**	**34.4**
**College graduate**	**42**	**17.8**
**Employment n = 236**		
**Yes**	**50**	**21.2**
**No**	**146**	**61.9**
**Retired**	**40**	**16.9**
**Monthly income n = 235** **<500**	**132**	**56.2**
**500**–**1000**	**57**	**24.3**
**> 1000**	**46**	**19.5**
**Menopausal status n = 236**		
**Premenopause**	**110**	**46.6**
**Perimenopause**	**36**	**15.3**
**Postmenopause**	**90**	**38.1**
**Pathological staging n = 134**		
**Stage 0 and I**	**40**	**29.9**
**Stage II**	**60**	**44.8**
**Stage III and IV**	**34**	**25.3**
**Distant metastasis n = 236**		
**Yes**	**17**	**7.2**
**No**	**219**	**92.8**
**Treatment modality n = 236**		
**Lumpectomy**	**121**	**51.3**
**Mastectomy**	**118**	**50.0**
**Lymph node dissection**	**200**	**85.1**
**Chemotherapy**	**190**	**80.5**
**Radiotherapy**	**198**	**83.9**
**Hormonal therapy**	**164**	**69.8**

### Profile of quality of life scale scores

Participants had a mean score for global health of 63.9 (95% CI 61.2-66.6). Among functional scales, social functioning scored the highest (77.5 [95% CI 73.65-81.38]) whereas emotional functioning scored the lowest (63.4 [95% CI 59.12-67.71]).

The most distressing symptom on the symptom scales was fatigability (Mean 35.2 [95% CI 31.38-39.18]) followed by sleep disturbance and pain (Table 2). Using the disease specific tool it was found that sexual functioning scored the lowest (Mean 25.9 [95% CI 70.23-77.90]) indicating poor functioning whereas body image scored the highest (Mean 75.64 [95% CI 71.79-79.48]). On the symptom scale, upset due to hair loss scored the highest (Mean 46.3 [95% CI 37.82-54.84]) indicating worse functioning followed by arm symptoms (Mean 36.58 [95% CI 32.50-40.65]).

**Table 2:** Mean score of all items in QLQ-C30 and QLQ-BR23 (N = 239)

**Variables**	**N**	**No. of items**	**Mean (SD)**	**95% CI**	**N (%) scoring <33.3)**^**a**^	**N (%) scoring ≥66.7)**^**a**^
**QLQ-C30**						
**Global health status/QoL**	**238**	**2**	**63.93(21.34)**	**61.21-66.66**	**13(5.4)**	**92(38.5)**
**Functional scales**^**b**^						
**Physical functioning**	**239**	**5**	**74.92(21.69)**	**72.15-77.68**	**9(3.8)**	**154(64.4)**
**Role functioning**	**237**	**2**	**68.84(35.96)**	**64.24-73.44**	**33(13.8)**	**132(55.2)**
**Emotional functioning**	**236**	**4**	**63.41(33.46)**	**59.12-67.71**	**52(21.8)**	**118(49.4)**
**Cognitive functioning**	**238**	**2**	**73.38(29.87)**	**69.57-77.20**	**24(10.0)**	**140(58.6)**
**Social functioning**	**238**	**2**	**77.52(30.27)**	**73.65-81.38**	**21(8.8)**	**152(63.6)**
**Symptom scales**^**c**^						
**Fatigue**	**239**	**3**	**35.28(30.62)**	**31.38-39.18**	**117(49.0)**	**39(16.3)**
**Nausea and vomiting**	**238**	**2**	**10.29(30.77)**	**6.36-14.22**	**208(87.0)**	**9(3.8)**
**Pain**	**238**	**2**	**29.97(31.23)**	**25.98-33.96**	**135(56.5)**	**31(13.0)**
**Dyspnoea**	**239**	**1**	**20.22(30.32)**	**16.35-24.08**	**149(62.3)**	**15(6.3)**
**Sleep disturbance**	**239**	**1**	**30.12(39.29)**	**25.11-35.13**	**136(56.9)**	**42(17.6)**
**Appetite loss**	**239**	**1**	**13.38(27.62)**	**9.86-16.90**	**185(77.4)**	**11(4.6)**
**Constipation**	**239**	**1**	**17.99(30.66)**	**14.08-21.89**	**163(68.2)**	**18(7.5)**
**Diarrhea**	**239**	**1**	**6.83(18.95)**	**4.41-9.24**	**205(85.8)**	**4(1.7)**
**Financial impact**	**239**	**1**	**34.58(42.26)**	**29.20-39.97**	**130(54.4)**	**57(23.8)**
**QLQ-BR23**						
**Functional scales**^**b**^						
**Body image**	**234**	**4**	**75.64(29.86)**	**71.79-79.48**	**24(10.0)**	**160(66.9)**
**Sexual functioning**	**234**	**2**	**25.92(29.77)**	**70.23-77.90**	**10(4.2)**	**129(54.0)**
**Sexual enjoyment**	**116**	**1**	**48.56(32.12)**	**45.52-57.34**	**16(6.7)**	**23(9.6)**
**Future perspective**	**236**	**1**	**61.29(39.37)**	**56.25-66.34**	**43(18.0)**	**105(43.9)**
**Symptom scales**^**c**^						
**Systemic side effect**	**236**	**7**	**19.27(17.76)**	**16.98-21.55**	**187(78.2)**	**4(1.7)**
**Breast symptoms**	**236**	**4**	**13.66(18.06)**	**11.34-15.98**	**195(81.6)**	**4(1.7)**
**Arm symptoms**	**236**	**3**	**36.58(31.76)**	**32.50-40.65**	**113(47.3)**	**34(14.2)**
**Upset by hair loss**	**100**	**1**	**46.33(42.87)**	**37.82-54.84**	**38(15.9)**	**32(13.4)**

^a^For functional scales, subjects scoring < 33.3% have problems; those scoring ≥ ⊠66.7% have good functioning. For symptom scales/symptoms, subjects scoring < 33.3% have good functioning; those scoring = 66.7% have problems.

^b^For functional scales, higher scores indicate better functioning.

^c^For symptom scales, higher scores indicate worse functioning.

### Factors associated with QoL scale scores

#### Global health and Functional scale in QLO-C30

There were significant differences in the global health means across categories of marital status (P =0.041), menopausal status (P =0.016), history of metastases (P = 0.016), monthly income (P =0.036) and type of surgery (P =0.026 and 0.017 for mastectomy and lumpectomy respectively). Post hoc analysis results revealed that subjects who were not married, premenopausal, with no history of metastases, have high income and who were treated by lumpectomy tended to have better global health related quality of life (Table 3).

**Table 3:** **Global health and functional scales in QLQ-C30 by independent variables (N = 239)**^**a**^

**Characteristic**	**Global health /QoL Mean(SD)**	**Functional scales in QLQ-C30**^**b**^				
		**Physical functioning Mean(SD)**	**Role functioning Mean (SD)**	**Emotional functioning Mean (SD)**	**Cognitive functioning Mean (SD)**	**Social functioning Mean (SD)**
Age						
≤50 year	67.7(20.04)	77.37(20.47)	69.97(37.06)	60.30(33.32)	70.31(30.42)	76.03(32.89)
>50 years	58.90(22.07)	71.63(22.91)	67.32(34.55)	67.57(33.37)	77.55(28.71)	79.53(26.33)
P-value	0.147	0.271	0.176	0.710	0.412	0.005
Time since diagnosis						
Early diagnosed	61.66(27.20)	74.09(24.02)	58.82(40.04)	55.00(38.57)	70.95(32.92)	70.47(39.41)
Transitional period	62.76(20.99)	73.69(21.30)	66.66(36.06)	64.37(33.02)	74.93(29.76)	76.37(28.90)
Long term survivors	67.00(18.65)	77.36(21.30)	76.97(32.54)	65.76(31.44)	71.92(28.83)	82.67(27.14)
P-value	0.478	0.400	0.034	0.461	0.574	0.179
Marital status						
Single	68.11(18.05)	73.91(23.69)	61.59(40.95)	59.78(39.22)	69.56(31.24)	66.66(41.43)
Married	65.56(19.97)	77.83(17.98)	70.96(34.17)	63.95(32.00)	74.84(28.92)	81.04(25.86)
Divorced	58.33(27.34)	62.85(29.31)	50.00(44.61)	55.95(38.31)	57.14(34.41)	70.23(37.65)
Widowed	56.04(24.45)	67.50(28.08)	69.58(36.57)	66.04(35.37)	75.41(30.65)	71.79(35.28)
P-value	0.041	0.123	0.216	0.780	0.205	0.371
Educational level						
Illiterate	61.25(24.29)	73.11(21.85)	67.77(33.59)	75.00(30.66)	78.24(28.74)	77.40(24.32)
Primary	54.16(25.21)	60.66(27.14)	57.77(42.82)	54.72(32.65)	75.55(30.86)	77.22(30.47)
Intermediate	68.56(23.13)	78.55(23.09)	64.39(42.81)	65.94(36.31)	71.73(33.87)	71.01(38.34)
High school	66.87(17.12)	79.17(18.63)	73.04(33.18)	59.77(34.25)	71.19(29.46)	79.21(33.17)
College graduate	65.27(65.27)	76.34(76.34)	70.73(70.73)	58.33(58.33)	69.44(69.44)	77.38(77.38)
P -value	0.059	0.009	0.548	0.009	0.284	0.535
Employment						
Yes	67.83(15.88)	76.53(21.81)	73.00(36.24)	60.20(32.46)	72.00(29.82)	78.00(31.66)
No	62.47(23.53)	73.51(21.89)	65.52(36.75)	64.00(33.58)	73.44(30.26)	74.48(30.68)
Retired	62.91(18.58)	76.83(21.08)	74.12(32.58)	64.37(35.45)	74.16(29.46)	87.08(26.00)
P-value	0.428	0.516	0.196	0.559	0.924	0.024
Monthly income						
<500	60.55(23.06)	71.46(23.62)	63.61(36.96)	62.05(34.81)	72.39(30.22)	71.75(32.61)
500–999	67.10(17.35)	78.59(18.88)	69.34(37.16)	65.02(32.54)	75.73(29.22)	79.82(30.00)
>1000	68.47(19.79)	79.13(17.92)	82.60(27.65)	64.13(32.00)	72.82(30.70)	90.94(17.46)
P-value	0.036	0.087	0.005	0.974	0.818	0.001
Menopausal status						
Premenopause	67.20(20.32)	76.84(20.34)	67.43(37.60)	59.49(34.30)	69.54(31.75)	74.24(33.45)
Perimenopause	67.82(16.07)	77.77(20.15)	71.75(35.37)	62.96(33.65)	69.90(30.03)	83.79(28.86)
Postmenopause	57.77(23.19)	70.88(23.51)	68.51(34.74)	67.97(32.41)	79.21(26.74)	78.65(26.58)
P-value	0.016	0.142	0.831	0.123	0.057	0.268
Pathological staging						
Stage 0 and I	66.87(17.65)	74.33(22.26)	66.66(36.98)	59.58(31.72)	69.58(32.66)	82.08(28.59)
Stage II	65.55(23.14)	74.55(23.43)	69.49(35.17)	64.58(32.92)	77.50(28.59)	72.31(30.88)
Stage III and IV	58. 08 (26.47)	68.23(23.60)	50.98(41.83)	53.67 (37.67)	67.15 (28.86)	65.68(34.55)
P-value	0.295	0.411	0.072	0.313	0.151	0.054
Metastases						
Yes	52.45(19.93)	55.29(24.69)	26.47(38.66)	42.64(37.71)	55.88(36.29)	57.29(37.00)
No	64.56(21.25)	76.22(20.76)	71.81(33.79)	64.89(32.77)	74.61(29.03)	78.84(29.41)
P value	0.016	0.001	0.000	0.016	0.024	0.009
Mastectomy						
Yes	60.47(22.93)	71.86(22.81)777.57(20.22)	64.22(37.31)	61.49(34.97)	74.64(28.91)	73.50(30.57)
No	66.87(19.22)		72.74(34.43)	65.02(32.18)	71.89(30.94)	81.21(29.84)
P-value	0.026	0.053	0.053	0.536	0.613	0.010
Lumpectomy						
Yes	67.08(19.26)	77.68(20.36)	72.86(34.16)	65.48(31.92)	71.90(30.58)	81.40(29.59)
No	60.14(22.88)	71.59(22.70)	63.86(37.59)	60.91(35.23)	74.70(29.26)	73.09(30.76)
P-value	0.017	0.033	0.048	0.435	0.514	0.007
Lymph node dissection						
Yes	63.44(21.19)	75.50(21.28)	67.92(36.40)	62.64(33.70)	73.11(29.94)	77.47(30.50)
No	65.00(22.75)	70.47(24.10)	70.95(34.61)	65.71(33.07)	73.33(30.30)	77.14(30.54)
P-value	0.771	0.255	0.753	0.545	0.937	0.776
Chemotherapy						
Yes	64.41(20.95)	76.00(20.73)	67.28(37.02)	61.31(34.19)	73.54(29.26)	77.16(30.70)
No	60.68(22.88)	69.42(24.88)	73.55(31.73)	71.19(30.00)	72.10(32.78)	78.26(29.36)
P-value	0.358	0.122	0.472	0.080	0.828	0.895
Radiotherapy						
Yes	64.25(21.06)	75.82(21.94)	69.72(36.11	63.77(33.12)	73.40(29.60)	79.10(29.48)
No	60.74(22.83)	68.94(19.68)	62.28(35.65)	60.58(36.25)	72.52(31.96)	68.42(33.73)
P-value	0.538	0.024	0.172	0.779	0.992	0.043

^a^P-value based on Kruskal Wallis or Mann Whitney tests.

^b^For functional scales, higher scores indicate better functioning.

Differences in the physical functioning means were observed across categories of educational level (P =0.009), history of metastases (P =0.001) and history of lumpectomy (P =0.033). Post hoc analysis showed that educated subjects who finished high school and had conservative breast surgery (lumpectomy) had better functioning on the physical scale.

#### Symptom scales in QLQ-C30

With the exception of financial impact, there were significant differences in all symptom scales across categories of metastasis. Post hoc analysis showed that women with metastases experienced worse symptoms. Differences in pain means were seen among age (P = 0.003), menopause (P = 0.003) and metastases categories (P = 0.001). Post hoc analysis revealed that younger, pre-menopausal women and those with a history of metastases experienced more pain.

#### Functional and symptom scales in QLQ-BR 23

Differences in means of body image were significant among categories of educational level (P = 0.029), and mastectomy (P = 0.022). Post hoc analysis showed that women who had undergone mastectomy and were highly educated tended to have poorer body image (Table 4). Better sexual functioning was observed for married women (P < 0.001), high income (P < 0.001), long term survivors (P = 0.027).

**Table 4:** **Functional and symptom scales in QLQ-BR23**^**a**^

	**Functional scales in BR 23**^**b**^				**Symptom scale in BR 23**^**c**^			
**Characteristic**	**Body Image**	**Sexual functioning**	**Sexual enjoyment**	**Future perspective**	**Systemic therapy side effect**	**Breast symptoms**	**Arm symptoms**	**Upset by hair loss**
Age								
≤50 year	72.53(31.24)	29.25(29.88)	48.91(34.02)	55.55(39.69)	20.07(18.66)	16.35(19.75)	39.09(31.74)	51.41(43.47)
>50 years	79.88(27.45)	21.38(29.16)	47.86(28.40)	68.97(37.78)	18.19(16.52)	10.06(14.87)	33.22(31.64)	39.02(41.43)
P-value	0.134	0.744	0.103	0.257	0.453	0.034	0.983	0.257
Time since diagnosis								
Early diagnosed	65.47(37.33)	18.09(27.52)	55.55(35.76)	44.76(44.23)	25.85(19.77)	20.95(22.17)	43.80(32.44)	70.83(34.15)
Transitional period	75.67(30.32)	23.65(27.71)	44.44(27.89)	63.22(39.06)	17.83(17.28)	11.11(14.45)	32.18(29.57)	41.49(42.23)
Long term survivors	80.33(23.83)	33.33(32.76)	52.27(36.22)	65.77(35.92)	18.59(17.10)	14.55(20.50)	40.59(34.19)	41.90(44.53)
P-value	0.292	0.027	0.338	0.037	0.065	0.044	0.093	0.035
Marital status								
Single	74.63(34.31)	5.07(16.99)	66.66(−)	60.86(44.55)	25.05(25.32)	13.40(24.71)	35.26(29.89)	33.33(40.20)
Married	73.88(29.97)	37.47(29.33)	48.83(32.03)	61.63(38.46)	16.96(14.81)	14.30(17.74)	38.22(31.81)	43.28(43.03)
Divorced	80.95(27.62)	4.76(12.10)	0.00(−)	52.38(46.61)	30.95(20.09)	18.45(16.72)	35.71(31.17)	86.66(32.20)
Widowed	81.25(27.59)	0.00 (0.00)	-	63.33(38.34)	20.95(20.51)	9.58(15.04)	31.11(33.30)	43.33(38.65)
P-value	0.446	0.000	0.288	0.877	0.060	0.046	0.484	0.020
Educational level								
Illiterate	82.32(27.79)	24.71(31.87)	52.56(30.07)	75.00(33.96)	16.06(14.95)	9.72(15.65)	32.03(31.36)	28.07(40.46)
Primary	78.88(25.49)	11.11(22.46)	33.33(30.86)	57.77(40.05)	21.11(17.58)	13.61(20.81)	43.70(34.39)	71.42(40.49)
Intermediate	71.01(33.32)	28.98(32.26)	52.77(30.01)	63.76(44.84)	17.59(20.47)	16.30(16.94)	35.26(34.26)	75.00(38.83)
High school/diploma	75.30(30.46)	26.95(27.20)	44.20(34.46)	55.14(39.84)	20.92(18.87)	15.12(18.49)	36.35(30.73)	40.47(41.99)
College	67.26(31.20)	34.52(31.96)	55.55(30.56)	54.76(38.83)	20.18(17.90)	15.07(18.88)	39.15(31.44)	55.07(42.17)
P -value	0.029	0.015	0.417	0.026	0.432	0.490	0.067	0.021
Employment								
Yes	76.19(28.31)	29.25(30.90)	53.33(34.69)	50.66(38.82)	20.47(19.76)	16.66(20.68)	38.00(30.12)	51.38(40.50)
No	75.57(30.84)	24.94(29.14)	47.29(31.69)	63.69(38.93)	18.42(17.08)	13.47(18.10)	35.31(32.35)	42.30(43.34)
Retired	75.20(28.77)	25.41(31.12)	47.05(31.31)	65.83(40.28)	20.83(17.80)	10.62(13.73)	39.44(32.11)	50.72(45.91)
P-value	0.885	0.697	0.756	0.070	0.683	0.425	0.610	0.558
Monthly income								
<500	75.38(30.40)	17.30(25.95)	42.85(30.42)	60.60(40.52)	21.51(17.70)	13.63(18.34)	35.35(30.44)	47.27(42.88)
500–999	75.00(29.37)	35.38(31.82)	50.00(35.30)	61.40(40.72)	17.62(20.91)	14.91(17.23)	37.42(33.89)	43.05(43.38)
>1000	78.62(27.97)	78.62(30.19)	55.95(30.16)	63.76(35.01)	15.21(12.32)	10.86(16.18)	37.68 (32.32)	48.33(45.20)
P-value	0.780	0.000	0.231	0.965	0.035	0.354	0.901	0.906
Menopausal status								
Premenopause	71.43(31.97)	30.00(29.93)	48.48(34.68)	56.36(39.29)	19.56(17.73)	17.27(20.05)	40.30(31.92)	48.97(43.08)
Perimenopause	77.31(28.28)	26.85(29.08)	49.01(26.66)	56.48(41.26)	20.50(20.22)	15.74(19.08)	34.87(32.00)	54.76(46.42)
Postmenopause	80.20(27.22)	20.45(29.33)	48.48(30.15)	69.25 (37.80)	18.408(16.88)	8.42(13.42)	32.71(31.31)	39.81(42.02)
P-value	0.091	0.049	0.985	0.047	0.876	0.001	0.169	0.473
Pathological staging								
Stage 0 and I	76.28(28.26)	22.64(26.34)	43.75(33.81)	60.68(41.09)	19.65(13.69)	17.09(18.72)	42.16(32.16)	56.25(48.25)
Stage II	78.67(29.57)	25.42(28.42)	47.31(33.08)	61.58(40.02)	20.33(19.24)	14.40(20.63)	36.34(31.21)	33.33(36.00)
Stage III and IV	61.51(36.17)	20.09(25.87)	48.88(27.79)	52.94(45.03)	24.36(21.51)	15.93(19.82)	36.92(34.46)	50.00(40.82)
P-value	0.052	0.761	0.884	0.609	0.691	0.560	0.363	0.225
Metastases								
Yes	71.56(35.36)	14.70(24.21)	33.33(36.51)	47.05(39.19)	36.13(29.26)	28.43(25.52)	54.24(35.54)	43.33(44.58)
No	75.96(29.46)	26.80(30.03)	49.39(31.84)	62.40(39.26)	17.95(15.91)	12.51(16.90)	35.21(31.12)	46.81(43.14)
P-value	0.585	0.117	0.299	0.131	0.013	0.008	0.033	0.831
Mastectomy								
Yes	70.01(33.22)	26.35(29.13)	47.77(32.10)	62.14(40.63)	19.69(19.06)	14.33(19.21)	37.75(31.66)	46.80(43.21)
No	81.26(24.97)	25.49(30.52)	49.40(32.40)	60.45(38.22)	18.84(16.41)	12.99(16.89)	35.40(31.95)	46.15(43.36)
P-value	0.022	0.665	0.768	0.684	0.964	0.827	0.499	0.965
Lumpectomy								
Yes	80.76(24.91)	24.58(30.47)	48.80(33.61)	60.60(38.49)	18.80(16.26)	13.42(17.98)	35.44(31.76)	45.28(43.40)
No	70.24(33.58)	27.33(29.09)	48.33(30.94)	60.02(40.43)	19.75(19.26)	13.98(18.22)	37.77(31.86)	47.82(43.12)
P-value	0.059	0.322	0.933	0.736	0.931	0.846	0.529	0.788
Lymph node dissection								
Yes	74.53(30.39)	25.75(29.59)	47.81(33.38)	59.50(39.52)	19.59(17.60)	13.12(17.15)	37.22(31.87)	48.23(43.18)
No	81.42(26.66)	25.71(30.87)	52.08(24.24)	70.47(37.72)	17.50(19.07)	15.71(22.11)	32.06(31.28)	38.46(42.70)
P-value	0.249	0.884	0.754	0.115	0.406	0.728	0.348	0.434
Chemotherapy								
Yes	73.89(30.73)	27.03(29.70)	48.14(33.40)	60.17(39.97)	20.10(18.26)	14.29(18.49)	37.95(31.22)	46.89(42.58)
No	82.78(25.05)	21.37(29.95)	50.98(23.91)	65.94(36.84)	15.76(15.15)	11.05(16.10)	30.91(33.69)	43.58(47.88)
P-value	0.071	0.161	0.781	0.387	0.144	0.213	0.097	0.806
Radiotherapy								
Yes	76.56(29.28)	26.14(30.12)	50.34(31.83)	61.11(39.27)	18.37(16.86)	13.25(17.79)	36.64(31.58)	43.20(42.30)
No	70.72(32.78)	24.77(28.22)	38.88(32.83)	62.28(40.39)	24.06(21.58)	15.78(19.54)	36.25(33.15)	61.11(44.64)
P-value	0.335	0.970	0.193	0.837	0.220	0.389	0.869	0.117

^a^P-value based on Kruskal Wallis or Mann Whitney tests.

^b^For functional scales, higher scores indicate better functioning.

^c^For symptom scales, higher scores indicate worse functioning.

More intense upset by hair loss was noted among women who were recently diagnosed (P = 0.035); divorced as opposed to single women (P = 0.020) and those who had intermediate education (P = 0.021).

Women who had metastases complained of more severe systemic side effects (P = 0.013), breast (P = 0.008) and arm symptoms (P = 0.033).

Women who were recently diagnosed were more worried about their future (P = 0.037), and complained of more breast symptoms (P = 0.044) and were more upset by the loss in their hair (P = 0.035).

### Predictors of quality of life

The predictors explained 24% of the variation in global health (R-squared = 0.24). The predictors which had a significant effect on global health given the other predictors in the model were staging of the disease (P = 0.005) and menopausal status (P = 0.031) (Table 5). The same model was built for every domain in QLQ-C30. Metastasis was a significant predictor in the physical and role functioning models (P = 0.002 and 0.003) respectively. Co-morbidities and chemotherapy were significant predictors in role functioning model (P = 0.032 and 0.009) respectively.

**Table 5:** Final linear regression model with parameter estimates for QLQ functional scales

	**Global QoL score**		**Physical functioning**		**Role functioning**		**Emotional functioning**		**Cognitive functioning**		**Social functioning**	
**Variable**	**Standardized Coefficients Beta**	**Significance**	**Standardized Coefficients Beta**	**Significance**	**Standardized Coefficients Beta**	**Significance**	**Standardized Coefficients Beta**	**Significance**	**Standardized Coefficients Beta**	**Significance**	**Standardized Coefficients Beta**	**Significance**
Constant	63.298	<0.001	36.082	0.024	56	0.032	77.93	0.001	71.909	0.001	41.898	0.047
**Age > 50**	−0.007	0.956	0.187	0.103	0.022	0.848	2.757	0.007	0.135	0.285	0	0.999
No = 0												
Yes = 1												
**Married**	0.116	0.231	0.129	0.168	0.076	0.434	0.837	0.404	0.11	0.288	0.35	<0.001
No = 0												
Yes = 1												
**Education**	−0.024	0.844	0.192	0.103	−0.021	0.861	−0.25	0.803	−0.112	0.384	−0.137	0.244
No = 0												
Yes = 1												
**Employment**	−0.018	0.853	−0.109	0.252	−0.078	0.429	−0.863	0.39	0.036	0.732	−0.054	0.571
No = 0												
Yes = 1												
**High Income**	0.109	0.29	0.086	0.391	0.171	0.098	1.612	0.11	0.101	0.361	0.25	0.014
No = 0												
Yes = 1												
**Menopause**	−0.259	0.031	−0.128	0.269	0.068	0.565	−0.621	0.536	−0.01	0.934	0.034	0.769
No = 0												
Yes = 1												
**Advanced stage**	−0.275	0.005	−0.061	0.516	−0.189	0.051	−0.54	0.59	−0.106	0.304	−0.156	0.101
No = 0												
Yes = 1												
**Late survivors**	−0.105	0.263	−0.021	0.819	0.071	0.447	0.861	0.391	−0.067	0.506	−0.015	0.873
No = 0												
Yes = 1												
**Comorbidities**	−0.027	0.783	−0.046	0.63	0.054	0.58	−2.171	0.032	0.037	0.725	−0.088	0.363
No = 0												
Yes = 1												
**Metastases**	−0.086	0.385	−0.297	0.002	−0.303	0.003	−1.691	0.094	−0.174	0.1	−0.103	0.285
No = 0												
Yes = 1												
**Lumpectomy**	0.321	0.205	0.45	0.068	0.15	0.551	0.746	0.457	0.072	0.789	0.265	0.282
No = 0												
Yes = 1												
**Mastectomy**	0.319	0.203	0.417	0.087	0.109	0.659	0.938	0.35	0.128	0.632	0.166	0.495
No = 0												
Yes = 1												
**Lymph node dissection**	−0.068	0.461	0.052	0.559	−0.01	0.917	−1.763	0.081	−0.013	0.892	0.031	0.731
No = 0												
Yes = 1												
**Chemotherapy**	−0.033	0.743	−0.07	0.466	−0.061	0.535	−2.646	0.009	−0.095	0.371	−0.043	0.654
No = 0												
Yes = 1												
**Radiotherapy**	−0.085	0.376	0.159	0.089	0.061	0.524	0.139	0.89	−0.046	0.65	0.083	0.377
No = 0												
Yes = 1												
**Hormonal therapy**	0.036	0.688	−0.063	0.464	−0.178	0.047	−0.76	0.449	0.04	0.677	0.008	0.931
No = 0												
Yes = 1												
**R squared**	0.24		0.28		0.25		0.25		0.132		0.281	
**P-value**	0.015		0.002		0.009		0.01		0.455		0.002	

## Discussion

This is the first study to assess quality of life of breast cancer survivors in Bahrain and indicates that Bahraini women with breast cancer have average to good quality of life functioning and low to average symptoms experience. Not surprisingly, the presence of metastases, advanced staging, having had a mastectomy as opposed to lumpectomy and the shorter time elapsed since diagnosis had a major effect across all the domains of quality of life of breast cancer survivors.

### Comparison with previous literature

Our results were largely comparable to other Western and Asian studies [13-15]. However, there are specific domains that showed lower scores which could be related to socio-cultural and religious aspects.

The global health score obtained in this study from Bahraini breast cancer survivors (63.9) is similar to that obtained in other Western and Asian studies such as South Korea (66.5), United Kingdom (66.8 and 69.8) and Germany (65.5) [13-15]. This study was also similar to other studies in Europe and Asia in showing that the poorest functioning in terms of symptoms was for fatigue followed by sleep disturbance, pain, hair loss and arm symptoms [10,13-19].

Within this region, Bahraini women with breast cancer have a lower quality of life than their counterparts in the United Arab Emirates (74.6) but higher than Kuwait (45.0) and Iran (32.0) [10,16,17]. However, caution has to be used in comparing data from these studies as the base populations vary in terms of age of participants, time elapsed since diagnosis and the staging of disease.

It is of note that global quality of life amongst Bahraini women was comparable with other studies despite the limited psychological support for breast cancer survivors in the Bahraini health care system. It may be that Bahraini women receive psychological support through other means such as the family or the wider society [20]. It could also be that participants in this study had greater difficulty understanding the meaning of quality of life and consequently responded to questions more positively.

There is a substantial body of literature documenting that comparison of quality of life data should go beyond the usual presentation of observed mean scores [12,21]. Various approaches have been recommended but so far there is no comprehensive approach suitable for the interpretation of quality of life results from a global perspective. Some of the suggested approaches are: using population-based reference values [12,22]; reporting the minimum important difference (mostly a difference of 10 points or more was used to define a clinically relevant change) [23]; and defining a particular proportion of patients achieving a predefined degree of benefit [23]. Although these methods are meaningful, they are arbitrary and subject to individual’s opinions. In this study we used 10 points as the minimum important difference and the proportion of patients achieving a particular degree of benefit as two methods of interpreting our quality of life data. For example, although the mean score for global QLQ – C30 indicated average to good functioning, only a third (38.5%) of participants met the 66.7% criterion for good functioning. Using the same criteria, poorer functioning for global quality of life was reported in Kuwait (10.9% scored ≥ 66.7 on the same scale) [17]. Problematic functioning for global quality of life in a Korean study was reported by 21.5% of participants [13], in Kuwait by 6.2% [17] and in our study by only 5.4%. This analysis is not available in many studies so comparison is not always possible. One should be cautious interpreting this finding because, while the sample in our study was chosen at random from a national cancer registry, the Korean study was hospital based and the Kuwaiti authors used a convenience sample. Another factor to mention is the higher mean age of our participants (50.2) compared to both studies (Range 46.6- 48.3).

Similar to many other studies [24,25], women showed an average performance on most functional scales except for sexual functioning and enjoyment which demonstrated poor functioning. Reasons suggested for disturbed sexual function include low self esteem, hair loss, abrupt menopause, vaginal dryness, partner's difficulty understanding one’s feelings and body image problems [24,25]. However, one should note that in our study most unmarried subjects did not respond to the question on sexual functioning as they may deem it culturally improper to express sexual desires or affairs “*And say to the believing women that they should lower their gaze and guard their modesty; that they should not display their beauty and ornaments except what (must ordinarily) appear thereof”(Sorat Al Noor 24:31, The Holy Quran)*. A similar argument was made in a Moroccan study that clearly described sexual impact in breast cancer as a taboo in the clinical setting [26].

### Factors associated with quality of life scores

The lack of an association between age and quality of life as opposed to most [15,18] but not all [19] previous studies could be due to several factors. First, different age groupings were used in the various studies. Second, the questionnaire does not contain questions about specific concerns related to younger women such as fertility and abrupt menopause [15,24], thereby reducing the impact of these issues on quality of life of younger women.

Interestingly, single women had better global quality of life, whereas married women had better physical functioning which is in agreement with some but not all studies [27,28]. One of the reasons may be related to the fact that single women are under less pressure to worry about their partner’s opinion because traditionally and religiously the local Islamic society places constraints around dating and premarital sex *“Nor come nigh to adultery: for it is a shameful (deed) and an evil, opening the road (to other evils”(Sorat Al Israa 17:32, The Holy Quran)*. On the other hand, polgyny is still allowed in some Islamic countries including Bahrain, with the specific limitation that a man can have up to four wives at any one time “*Marry women of your choice, Two or three or four; but if you fear that you shall not be able to deal justly (with them), then only one” (Sorat Al Nissa 4:3, The Holy Quran)*. This may be intimidating to some married women who fear that a serious and crippling illness could be an excuse for their husband to take a second wife, especially if the woman was unable to attend to her husband’s needs . Married women, however, functioned better physically as they had to continue to do the house work regardless of the disease [29].

Breast conservative surgery (lumpectomy) was not only associated with better global quality of life but also with better physical, role and social functioning as in previous studies [19,30]. Together with recent data about comparable survival time for both procedures in early stage breast cancer [31], this should have an implication on surgeon’s and patient’s choice of surgery. However, receiving chemotherapy, radiotherapy or hormone therapy was not associated with significant deterioration of quality of life. A significant amount of literature has shown that the impairment in quality of life due to such therapy is minor and limited to short term rather than long term quality of life [32,33].

Long term survivors showed better role functioning, sexual functioning and future perspectives compared to early survivors. On the other hand, early survivors reported more breast symptoms and were more upset by their hair loss. This is expected as the first year is usually the year during which patients receive adjuvant therapy and suffer from its various complications. This is in line with many previous studies which showed that the longer time since diagnosis is, the better the quality of life will be [18,34-36]. One should note that this study did not compare quality of life of the same individuals at several time intervals but compared different subjects with various time elapsed since diagnosis.

The current study provided important information about Bahraini breast cancer survivors with several strengths including randomized sampling method, use of standardized measures of quality of life, a satisfactory response rate of 71%, and the use of a clinically meaningful analysis. However, it has limitations that should be addressed in future research including lack of a disease-free control group and incomplete clinical information about cancer in the Cancer Registry especially with respect to grade and stage of the disease.

### Implications for practice and policy

The results are important when counseling patients about side effects of the disease and the need for greater attention to cancer related symptoms such as fatigue, pain, insomnia, arm symptoms and hair loss. Furthermore, sexual issues after breast cancer diagnosis and treatment should be addressed and explored in a culturally sensitive way. Due to improved quality of life and comparable survival time, lumpectomy should be considered in all women with early stage disease. Special care and attention should be given to women with metastatic lesions as their quality of life is markedly affected in most quality of life domains. Further research should address cultural differences in issues related to sexuality, body image and interpretation of quality of life as a concept.

## Conclusion

Bahraini breast cancer survivors reported favorable overall global quality of life. Bahraini women showed good functioning on most QLQ-C30 functional scales, with the lowest score for emotional functioning. Fatigue, sleep disturbance and pain were the most bothersome symptoms. In the disease specific tool, women reported the lowest performance in sexual enjoyment and functioning whereas arm symptoms and hair loss were among the most severe symptoms reported. Many factors were related to lower global quality of life including marital status, menopausal status, metastases, monthly income and type of surgery performed. Predictors of global health quality of life were staging of the disease and menopausal status whereas metastases predicted physical and role functioning. This study highlights the women at risk of poorer quality of life after breast cancer and the issues that most need to be addressed in this Middle East society.

## Abbreviations

EORTC QLQ-C30: European Organization for Research and Treatment of Cancer Quality of Life Cancer Specific Version; BR23: Quality of life breast cancer specific version; TNM: Tumor, lymph node, metastases; SPSS: Statistical Package for Social Sciences; ANOVA: Analysis of variance; QoL: Quality of life.

## Competing interests

The authors declare that they have no competing interests.

## Authors’ contributions

GJ participated in the design of the study, performed data collection and analysis and drafted the manuscript. DW participated in the design of the study, revised and helped to draft the manuscript. All authors read and approved the final manuscript**.**

## Pre-publication history

The pre-publication history for this paper can be accessed here:

http://www.biomedcentral.com/1471-2407/13/212/prepub
